# 603 Timing of autologous spray cell suspension: better early than late

**DOI:** 10.1093/jbcr/irac012.231

**Published:** 2022-03-23

**Authors:** Spogmai Komak

**Affiliations:** UT MD Anderson, Galveston, Texas

## Abstract

**Introduction:**

Autologous spray cell suspension is associated with multiple beneficial outcomes, however, the optimal timing of use has not been determined. We examined the timing of spay cell application, and hypothesize that early (< 72 hr) use is associated with faster definitive wound coverage, impacting multiple clinical outcomes compared to late ( >72 hr) use of spray cells.

**Methods:**

A retrospective review of 28 patients with spray cell application from March 2020 -- May 2021 was conducted. Pediatric patients < 16 yrs (n=1) as well as deaths (n=2--including 1 withdrawal) were excluded, leaving 25 patients. Those that received spray cells early (< 72 hrs, n=14) were compared to late ( >72 hrs, n=11). Time to index operation, time to spray cell application, rate of complete wound coverage, number of OR trips required for wound closure, wound infection rates, LOS / %TBSA, and overall hospital LOS were examined.

**Results:**

There was no difference in demographics or % TBSA burn (21.4% vs. 23/5%), between early and late spray cell groups. The early group had a significantly faster time to index operation (1.2 dys vs. 2.5 dys P= .05), and faster time to spray cell application (1.6 dys vs. 18.1 dys P=.036) (**Table 1).** The early group had a greater wound coverage rate at index operation —64.2% complete coverage vs. 18.1% in the late group (p< .03). The total number of OR trips needed for wound coverage was less in the early group (1.5 trips / pt) vs. the late (7.0 trips / pt) P=0.03. The early group had less wound infections, a shorter LOS / % TBSA burn ( 0.83 dys vs. 1.85 dys P=0.005), and shorter overall LOS (16.5 dys vs. 46.9 dys P=0.03).

**Conclusions:**

Autologous spray cell use has multiple beneficial outcomes, however, the optimal timing of application has not been determined. We report that early (< 72 hr) application of spray cells is associated with greater rate of complete wound coverage at index operation, decreased total number of operations needed for wound coverage, less wound infections, shorter LOS / %TBSA, and shorter overall LOS. This is likely related to earlier definitive wound coverage and closure, which is afforded by the large expansion ratio of spray cells.

